# Identification of Protective Amino Acid Metabolism Events in Nursery Pigs Fed Thermally Oxidized Corn Oil

**DOI:** 10.3390/metabo13010103

**Published:** 2023-01-08

**Authors:** Yue Guo, Lei Wang, Andrea Hanson, Pedro E. Urriola, Gerald C. Shurson, Chi Chen

**Affiliations:** 1 Department of Food Science and Nutrition, University of Minnesota, 1334 Eckles Ave., St. Paul, MN 55108, USA; 2 Department of Animal Science, University of Minnesota, 1364 Eckles Ave., St. Paul, MN 55108, USA

**Keywords:** oxidized lipids, amino acid metabolism, nursery pig, tryptophan-NAD^+^ biosynthesis, glutathione metabolism, threonine catabolism

## Abstract

Feeding thermally oxidized lipids to pigs has been shown to compromise growth and health, reduce energy digestibility, and disrupt lipid metabolism. However, the effects of feeding oxidized lipids on amino acid metabolism in pigs have not been well defined even though amino acids are indispensable for the subsistence of energy metabolism, protein synthesis, the antioxidant system, and many other functions essential for pig growth and health. In this study, oxidized corn oil (OCO)-elicited changes in amino acid homeostasis of nursery pigs were examined by metabolomics-based biochemical analysis. The results showed that serum and hepatic free amino acids and metabolites, including tryptophan, threonine, alanine, glutamate, and glutathione, as well as associated metabolic pathways, were selectively altered by feeding OCO, and more importantly, many of these metabolic events possess protective functions. Specifically, OCO activated tryptophan-nicotinamide adenosine dinucleotide (NAD^+^) synthesis by the transcriptional upregulation of the kynurenine pathway in tryptophan catabolism and promoted adenine nucleotide biosynthesis. Feeding OCO induced oxidative stress, causing decreases in glutathione (GSH)/oxidized glutathione (GSSG) ratio, carnosine, and ascorbic acid in the liver but simultaneously promoted antioxidant responses as shown by the increases in hepatic GSH and GSSG as well as the transcriptional upregulation of GSH metabolism-related enzymes. Moreover, OCO reduced the catabolism of threonine to α-ketobutyrate in the liver by inhibiting the threonine dehydratase (TDH) route. Overall, these protective metabolic events indicate that below a certain threshold of OCO consumption, nursery pigs are capable of overcoming the oxidative stress and metabolic challenges posed by the consumption of oxidized lipids by adjusting antioxidant, nutrient, and energy metabolism, partially through the transcriptional regulation of amino acid metabolism.

## 1. Introduction

Recycled oils and fats from deep frying and thermal processing practices in the food and feed industry have been widely used as an economical source of energy and lipids in livestock feeds [1,2]. However, the unfavorable chemical and organoleptic properties of these heavily oxidized lipids, including rancidity, elevated acidity, and increased lipid oxidation products (LOPs) [3], can impair growth performance, meat quality, and health of production animals, especially in rapidly growing young pigs [4,5,6]. For example, the levels of unsaturated aldehydes, which are major secondary LOPs in heavily oxidized lipids, had negative correlations with growth performance and feed intake in young pigs [7]. These detrimental effects on growth performance have been associated with compromised physiological functions, such as dysfunctions of the small intestine, thyroid hormone disorder, and altered concentrations of serum cytokines [4,6,8,9].

Nutrient metabolism, as an indispensable component of animal growth, is an expected target of dietary oxidized lipids to elicit their detrimental impacts on growth performance and health. The most well-known effect of feeding oxidized lipids on nutrient metabolism is the disrupted lipid metabolism in pigs and other species [4,10,11], because oxidized fatty acids can integrate into acylglycerols and phospholipids after absorption and regulate fatty acid oxidation and lipid synthesis through transcriptional regulation [12,13,14,15,16,17]. In contrast, the influence of feeding oxidized lipids on amino acid metabolism remains largely unexplored, partially due to the fact that the inclusion of oxidized lipids does not directly affect the amino acid and protein composition of the diet. However, dietary oxidized lipids have the potential to affect amino acid metabolism by both direct chemical interactions and indirect regulatory signaling. For the direct chemical interactions, unsaturated aldehydes in oxidized oils can react with free amino acids or basic amino acid residuals in protein, such as lysine, to form adducts through Schiff bases and Amadori rearrangement [18,19]. These reactions reduce the bioavailability of targeted amino acids as well as the digestibility and functions of affected proteins [20]. Moreover, the reactions between electrophilic LOPs and thiol-containing antioxidants elicit oxidative stress and imbalance in the antioxidant system [21]. As for the indirect regulation, oxidized fatty acids in frying oils, such as cyclic fatty acids, can function as the ligands of peroxisome proliferator-activated receptor alpha (PPARα), which is a regulator of amino acid metabolism [22], while lipidic aldehydes can affect the functions of the mammalian target of rapamycin (mTOR), a central regulator of protein metabolism [23]. The effects of feeding oxidized lipids on amino acid metabolism were observed in our mouse study, in which feeding oxidized soybean oil upregulated the hepatic tryptophan-nicotinamide adenine dinucleotide (NAD^+^) pathway and decreased serum tryptophan concentration [24]. However, the effects of feeding oxidized lipids on the amino acid metabolism in pigs and their underlying mechanisms are not well studied.

In our recent study, feeding three levels of oxidized corn oil (OCO) to nursery pigs for 35 days tended to decrease body weight gain but not feed intake compared with feeding the control corn oil (CCO) diet. Targeted analysis of serum metabolites showed dose-dependent decreases in tryptophan and α-tocopherol concentrations [25], but the influences of feeding OCO on the metabolic events related to amino acid and redox homeostasis were not examined. In the current study, comprehensive profiling of amino acids and their metabolites in the serum and liver samples from these nursery pigs fed OCO were conducted using liquid chromatography-mass spectrometry (LC-MS)-based untargeted metabolomic analysis. The OCO-responsive events and pathways in amino acid metabolism were further characterized by targeted metabolite and biochemical analyses for defining their roles in the tolerance and susceptibility of nursery pigs to the oxidized lipids in feed.

## 2. Materials and Methods

### 2.1. Chemicals and Reagents

The chemicals and reagents used in sample preparation, LC-MS analysis, structural confirmation, and quantification are provided in the supplementary material Table S1.

### 2.2. Animals, Experimental Design, and Sample Collection

The protocol of the feeding trial was reviewed and approved by the University of Minnesota Institutional Animal Care and Use Committee. The procedures of OCO preparation, diet formulation, and animal management have been described previously [25]. Briefly, OCO was prepared by heating CCO at 185 °C for 12 h with an airflow rate of 12 L/min. The feeding trial was conducted at the University of Minnesota Southern Research and Outreach Center (Waseca, MN, USA). A total of 128 barrows weaned at 19 days of age, with average initial body weight (BW) = 6.3 ± 1.4 kg, were assigned to 8 blocks (4 pens/block and 4 pigs/pen) based on their BWs. Pigs had ad libitum access to feed and water. Four pens in each block were assigned randomly to 4 isocaloric dietary treatments containing 9% CCO (as CCO control group), 6% CCO + 3% OCO (as 3% OCO group), 3% CCO + 6% OCO (as 6% OCO group), and 9% OCO (as 9% OCO group), respectively. The pig with the BW closest to the mean pen BW on day 0 of feeding was selected as the focal pig in each pen (n = 32). On day 35 of feeding, total 32 focal pigs (8 pigs/group) were euthanized, and blood and liver samples were collected under fed status. Serum was isolated after centrifugation, and tissue samples were snap-frozen and stored at −80 °C for metabolite and biochemical analysis.

### 2.3. LC-MS-Based Metabolomic Analysis

The procedures of sample preparation, sample analysis, and data analysis in metabolomics analysis used in this study were as follows.

#### 2.3.1. Sample Preparation

Liver tissue samples were fractionated using a modified Bligh and Dyer method [26]. Briefly, 100 mg of liver sample was homogenized in 0.5 mL of methanol and then mixed with 0.5 mL of chloroform and 0.4 mL of water. After 10 min centrifugation at 18,000× *g*, the upper aqueous fraction was harvested for hydrophilic metabolite analysis, and the organic fraction was dried under nitrogen and reconstituted in 0.5 mL *n*-butanol.

Serum samples were deproteinized and extracted by mixing 1 volume of serum with 19 volumes of 66% aqueous acetonitrile (ACN) and then centrifuged at 18,000× *g* for 10 min to obtain the supernatants.

#### 2.3.2. Chemical Derivatization

For detecting the metabolites containing amino functional groups in their structures, the samples were derivatized with dansyl chloride (DC) prior to the LC-MS analysis. Briefly, 5 µL of sample or amino acid standard was mixed with 5 µL of 50 µM *d*_5_-tryptophan as the internal standard, 50 µL of 10 mmol/L sodium carbonate, and 100 µL 3 mg/mL DC in acetone. The mixture was incubated at 60 °C for 15 min and centrifuged at 18,000× *g* for 10 min, and the supernatant was transferred into an HPLC vial for LC-MS analysis.

For detecting metabolites containing carboxyl group, the samples were derivatized with 2-2’-dipyridyl disulfide (DPDS), triphenylphosphine (TPP), and 2-hydrazinoquinoline (HQ) prior to the LC-MS analysis [27]. Briefly, 2 µL of sample or standard was added into 100 µL of freshly prepared ACN solution containing 1 mM DPDS, 1 mM TPP, 1 mM HQ, and 100 µM deuterated *d*_4_-acetic acid as the internal standard. The reaction mixture was incubated at 60 °C for 30 min, chilled on ice, and mixed with 100 µL of H_2_O. This mixture was centrifuged at 18,000× *g* for 10 min, and the supernatant was transferred into an HPLC vial for LC-MS analysis.

#### 2.3.3. Conditions of LC-MS Analysis

A 5 µL of aliquot prepared from serum and hepatic extracts was injected into an Acquity ultra-performance liquid chromatography-quadrupole time-of-flight mass spectrometry (UPLC-QTOFMS) system (Waters, Milford, MA, USA) and separated in a UPLC column in a 10 min run at a flow rate of 0.5 mL/min. Detailed information on LC-MS acquisition conditions is provided (Table S2). The LC eluant was injected into a Xevo-G2-S QTOF mass spectrometry for accurate mass measurement and ion counting. Capillary voltage and cone voltage for electrospray ionization was maintained at 3 kV and 30 V for positive mode detection or at −3 kV and −35 V for negative mode detection, respectively. Source temperature and desolvation temperature were set at 120 °C and 350 °C, respectively. Nitrogen was used as both cone gas (50 L/h) and desolvation gas (600 L/h), and argon was used as collision gas. For accurate mass measurement, the mass spectrometer was calibrated with sodium formate solution with a range *m*/*z* of 50–1000 and monitored by the intermittent injection of the lock mass leucine encephalin ([M + H]^+^ = 556.2771 *m*/*z* and [M−H]^−^ = 554.2615 *m*/*z*) in real-time. Mass chromatograms and mass spectral data were acquired and processed by MassLynx^TM^ 4.2 software (Waters) in centroided format.

#### 2.3.4. Targeted Quantitative Analysis

The concentrations of amino acids, NAD^+^, adenosine, adenosine monophosphate (AMP), inosine, α-ketobutyrate, α-aminobutyrate, glutathione (GSH), oxidized glutathione (GSSG), pyroglutamate, ascorbic acid, cystathionine, S-adenosyl-homocysteine (SAH), and S-adenosyl-methionine (SAM) were determined by calculating the ratio between their individual peak areas and the peak area of internal standard. Individual metabolite concentrations were determined by fitting the ratio between the peak area of each metabolite and the peak area of the internal standard with a standard curve using QuanLynx^TM^ 4.2 software (Waters).

#### 2.3.5. Untargeted Multivariate Data Analysis and Marker Characterization

After data acquisition in the UPLC-QTOFMS system, chromatographic and spectral data of samples were deconvoluted by MarkerLynx^TM^ 4.2 software. A multivariate data matrix containing information on sample identity, ion identity from retention time (RT) and *m/z*, and ion abundance was generated through centroiding, deisotoping, filtering, peak recognition, and integration. The intensity of each ion was calculated by normalizing the single ion counts (SIC) versus the total ion counts (TIC) in the whole chromatogram. The processed data matrix was exported into SIMCA-P+^TM^ 12 software (Umetrics, Kinnelon, NJ, USA). The partial least squares-discriminant analysis (PLS-DA) on CCO-, 3% OCO-, 6% OCO-, and 9% OCO-fed pigs as well as the orthogonal projections to latent structures discriminant analysis (OPLS-DA) between CCO- and all OCO-fed pigs were conducted after the data were transformed by mean-centering and *Pareto* scaling. Major latent variables of the multivariate model were defined in a scores scatter plot by a PLS-DA model. The potential metabolite markers were identified by analyzing ions contributing to the principal components and to the separation of sample groups in the loadings scatter plot by an OPLS-DA model.

The chemical identities of interested compounds were determined by accurate mass measurement, elemental composition analysis, and database search using Human Metabolome Database (https://www.hmdb.ca/, accessed on 1 December 2022), Kyoto Encyclopedia of Genes and Genomes (KEGG), Metlin (https://metlin.scripps.edu/, accessed on 1 December 2022), and comparisons with authentic standards if available.

### 2.4. Gene Expression Analysis

Total RNA from liver tissue was isolated using Trizol reagent from Invitrogen Life Technologies (Carlsbad, CA, USA) according to the manufacturer’s protocol. The cDNA was generated from 1 µg of total RNA using the High-Capacity cDNA Reverse Transcription Kit from Thermo Fisher Scientific (Wilmington, DE, USA). The sequence of the primers used for the quantitative real-time polymerase chain reaction (qPCR) analysis of targeted genes was enlisted (Table S3), including tryptophan 2,3-dioxygenase (*TDO*), indoleamine 2,3-dioxygenase (*IDO2*), kynureninase (*KYNU*), 3-hydroxyanthranilate 3,4-dioxygenase (*HAAO*), quinolinate phosphoribosyltransferase (*QPRT*), glutamate-cysteine ligase catalytic subunit (*GCLC*), glutathione-disulfide reductase (*GSR*), glutathione peroxidase 1 (*GPX1*), glutathione s-transferase (*GSTA*), and microsomal glutathione s-transferase 1 (*MGST1*). The expression levels of targeted genes were measured by qPCR using SYBR Green PCR Master Mix in a StepOne Plus system from Applied Biosystems (Carlsbad, CA, USA). The expression levels of genes were quantified using the comparative threshold cycle (CT) method with β-actin (*ACTB*) as the reference gene.

### 2.5. In Vitro Analysis of Threonine Dehydrogenase (TDG) and Threonine Dehydrotase (TDH) Enzymatic Activities

The hepatic TDG enzyme activity was determined in total 32 individual liver samples using a threonine dehydrogenase assay kit from Biomedical Research Service & Clinical Application (Buffalo, NY, USA). Briefly, the liver sample was homogenized with lysis buffer. The homogenate was centrifuged at 14,000× *g* for 5 min at 4 °C. The supernatant was harvested and stored at −80 °C for further analysis. Before starting the enzyme reaction assay, lysate liver protein concentration was determined using a Pierce^TM^ BCA Protein Assay Kit (Thermo Fisher Scientific, Waltham, MA, USA). The lysate liver samples were further diluted to a recommended range of 0.5–2 mg/mL. For determining the enzyme activity of TDG in CCO- or OCO-treated pigs, 10 µL of liver homogenates were mixed with either 50 µL reaction mix or blank in a 96-well plate. The plate was covered and incubated at 37 °C for 1 h. After the incubation, 50 µL of 3% acetic acid was used to stop the reaction. A cherry red color change was measured by a Spectra Max 250 96-well plate reader from Molecular Devices (Sunnyvale, CA, USA) at 492 nm. The TDG activity was defined in IU/L = ΔO.D. × 16.98.

For determining the influence of OCO on TDH enzyme activity, the TDH activity was indicated by measuring the production of its downstream metabolite concentration in an oil extracts coincubation enzyme reaction assay. Pig liver homogenate was prepared using a modified method from Bird and Nunn [28]. Briefly, the liver sample was homogenized in ice-cold 0.1 M KH_2_PO_4_ buffer (pH 8.2, 2.5 vol/wt). The homogenate was centrifuged at 42,000× *g* for 1 h at 4 °C, and the supernatant was collected. Hydrophilic compounds of CCO and CCO were extracted by mixing oils with 90% isopropanol 1:1 *v*/*v*. After vortexed and centrifuged at 18,000× *g* for 10 min, the aqueous phase was taken for further enzyme reaction assay. In the reaction assay, a 20 µL of liver homogenate was mixed either with CCO (20 µL CCO extract), OCO (20 µL OCO extract), or solvent blank (20 µL 90% isopropanol), with reaction mix containing 0.1 M KH_2_PO_4_ (pH 8.2), 1 mM dithiothreitol (DTT), and 0.2 mM pyridoxal 5’-phosphate (P-5-P). The reaction started by adding threonine as the substrate to a final concentration of 0.2 M. The mixture was incubated at 37 °C for 30 min. After the incubation, the reaction was stopped by adding ACN (10:1 *v*/*v*) to the reaction mix. The level of α-ketobutyrate production in the reaction system was measured by LC-MS targeted analysis that indicates the TDH activity. Three replicates were conducted for the enzyme reaction assay.

### 2.6. Statistical Analysis

Statistical analysis of metabolomics parameters, gene expression levels, and in vitro enzymatic assay was performed by one-way ANOVA followed by Tukey’s *post hoc* test, in which the CCO group was considered as the control group and compared to 3%, 6%, and 9% OCO treatment groups using GraphPad Prism version 8.0.2 (La Jolla, CA, USA). Correlation between serum and hepatic free amino acids was calculated by Pearson correlation using GraphPad Prism version 8.0.2 (La Jolla, CA, USA). Results are presented as mean ± standard error mean (SEM). Differences between dietary treatments were considered significant if *p* < 0.05 and were considered a trend if the *p*-value was between 0.05 and 0.10.

## 3. Results

### 3.1. Influences of OCO on Serum-Free Amino Acids

Blood amino acid concentration directly indicates the distribution and homeostasis of total amino acids after intake from the diet. To determine the influence of feeding OCO on blood amino acid distribution, a comparison of serum free amino acids in the nursery pigs fed three doses of OCO vs. CCO was conducted using quantitative analysis and multivariate modeling. The results of quantitative analysis showed that OCO selectively and dose-dependently affected serum free amino acids, causing decreases in the concentrations of alanine, glutamate, and tryptophan after feeding the 9% OCO diet, while increasing the percentage of threonine compared to CCO diet (Table 1).

**Figure 1:**
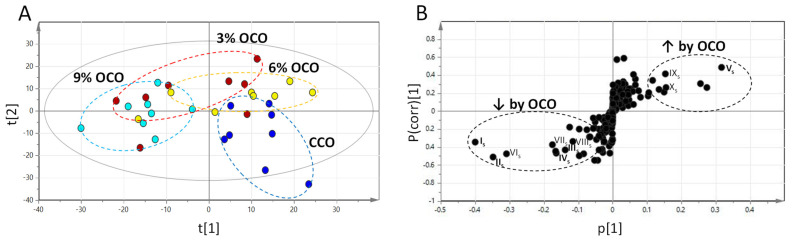
Profiling oxidized corn oil (OCO)-induced changes in serum amino acids. Data from LC-MS analysis of serum samples were processed by multivariate modeling. (**A**) Scores plot of the PLS-DA model on serum samples. (**B**) S-plot of the OPLS-DA model on serum samples. The identified OCO-responsive metabolites are labeled (I_s_–X_s_).

Multivariate modeling from the PLS-DA of detected amino-containing metabolites in serum further showed the dose-dependent separation among CCO and OCO treatments in a scores plot (Figure 1A). Major metabolites contributing to the separation between CCO and OCO were identified in a S-plot of the OPLS-DA model (Figure 1B). In addition to confirming the decreases in alanine (I_s_), glutamate (II_s_), and tryptophan (III_s_) and the increase in threonine (V_s_) in the quantitative analysis, the modeling also revealed a decrease in carnosine (IV_s_) from feeding OCO diets (Table 2). The concentration of serum carnosine was further quantified to validate this observation (Table 1). Other metabolites responsive to OCO (IV_s_–X_s_) were also identified using accurate mass-based database search (Table S4).

### 3.2. Influences of OCO on the Hepatic Metabolome

**Figure 2:**
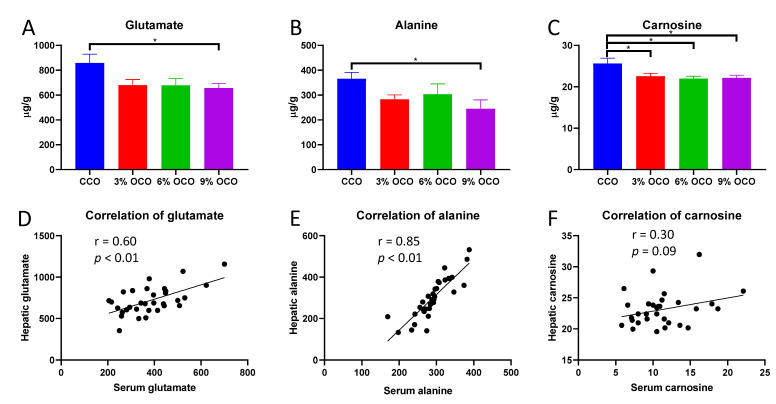
Concentrations of oxidized corn oil (OCO)-responsive amino acids in the liver and their correlations with serum concentrations. Hepatic concentrations of glutamate (**A**), alanine (**B**), and carnosine (**C**). Value = mean ± SEM (n = 8 pigs/group). * *p* < 0.05 from one-way ANOVA followed by the Tukey *post hoc* test between control corn oil (CCO) and OCO treatments. The correlations between hepatic and serum concentrations of glutamate (**D**), alanine (**E**), and carnosine (**F**) and their significances were calculated by Pearson correlation analysis.

Following the observations of the changes in serum amino acid profile, hepatic free amino acids were quantified to examine the correlations between these two pools of free amino acids (Table S5). Feeding OCO decreased glutamate, alanine, and carnosine concentrations (Figure 2A–C) but did not affect tryptophan, threonine, and other amino acid concentrations in the liver (Table S5). The correlation analysis on the concentrations of free amino acids in serum and liver revealed strong positive correlations in glutamate (Figure 2D), alanine (Figure 2E), and proline (Table S5) and a trend of correlation in carnosine (Figure 2F) but no correlations in other amino acids (Table S5).

**Figure 3:**
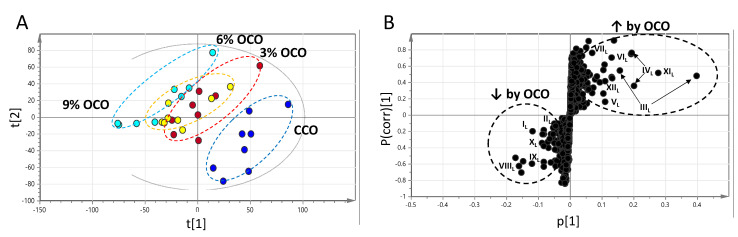
Influences of feeding oxidized corn oil (OCO) on hepatic metabolome. Data from LC-MS analysis of liver extractions were processed by PLS-DA modeling. (**A**) Scores plot of the PLS-DA model on liver samples. (**B**) S-plot of the OPLS-DA model on liver samples. The OCO-responsive metabolites are labeled (I_L_–XII_L_).

To further determine the metabolic events that could contribute to the OCO-elicited changes in free amino acids, the metabolomes in the polar hepatic extracts of CCO- and OCO-fed pigs were compared by the comprehensive LC-MS analyses using four different methods. The scores plot of the hepatic metabolome in the PLS-DA model showed a dose-dependent separation between CCO and OCO samples (Figure 3A). Major metabolites contributing to this separation (I_L_–X_L_) were identified in the S-plot of the OPLS-DA model (Figure 3B). Among the structurally confirmed hepatic metabolites contributing to the separation, NAD^+^ (VI_L_), AMP (III_L_), GSH (V_L_), GSSG (IV_L_), and pyroglutamic acid (VII_L_) were increased by feeding OCO, while alanine (II_L_) and ascorbic acid (I_L_) were decreased by feeding OCO diets (Table 3). Other hepatic metabolites responsive to dietary OCO (VIII_L_–XII_L_) are also identified using accurate mass-based database search (Table S6).

### 3.3. Effects of OCO on the Hepatic Tryptophan-NAD^+^ Pathway

**Figure 4:**
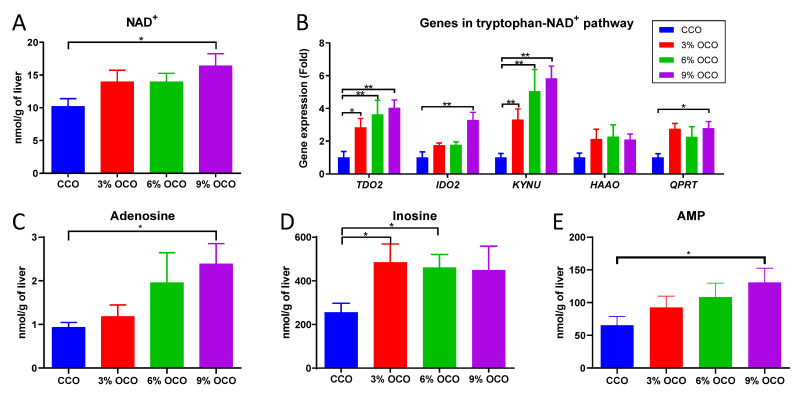
Effects of dietary concentration of oxidized corn oil (OCO) on the concentrations of metabolites and the levels of gene expression in the hepatic tryptophan-NAD^+^ pathway. (**A**) NAD^+^. (**B**) Gene expression levels of the enzymes in kynurenine pathway. The average gene expression level of control corn oil (CCO) liver samples was artificially set as 1. (**C**) Adenosine. (**D**) Inosine. (**E**) AMP. Value = mean ± SEM (n = 8 pigs/group). * *p* < 0.05 and ** *p* < 0.01 from one-way ANOVA followed by the Tukey *post hoc* test between CCO and OCO treatments.

Quantification of NAD^+^ in the liver confirmed that it was increased in a dose-dependent manner by feeding OCO (Figure 4A), which is in contrast with the decrease in serum tryptophan (Table 1), the precursor of the nicotinamide moiety in NAD^+^ from the kynurenine pathway of tryptophan metabolism. The transcriptional levels of the enzymes in the kynurenine pathway were also determined, and results showed that dietary OCO increased the expression levels of *TDO2*, *IDO2*, *KYNU*, and *QPRT* genes in a dose-dependent manner (Figure 4B). In addition, the metabolites related to the adenine dinucleotide moiety of NAD^+^, including adenosine, inosine, and AMP, were also increased by feeding OCO (Figure 4C–E).

### 3.4. Effects of OCO on GSH Metabolism

**Figure 5:**
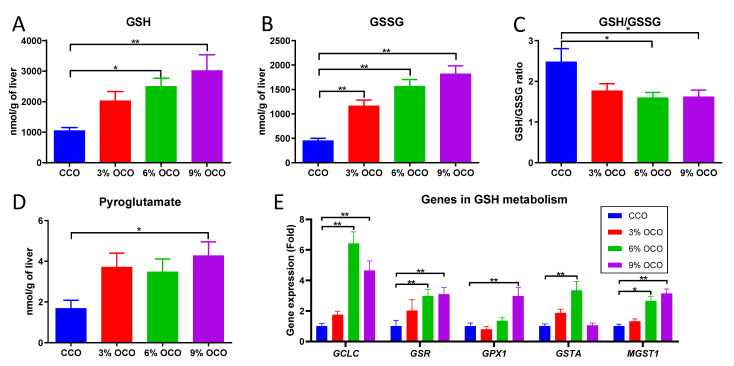
Effects of dietary concentrations of oxidized corn oil (OCO) on the concentrations of metabolites and the levels of gene expression in hepatic glutathione metabolism. (**A**) GSH. (**B**) GSSG. (**C**) GSH/GSSG ratio. (**D**) Pyroglutamate. (**E**) Gene expression level of the enzymes in GSH metabolism. The average expression level of genes in control corn oil (CCO) treatment of pig liver samples was artificially set as 1. Value = mean ± SEM (n = 8 pigs/group). * *p* < 0.05 and ** *p* < 0.01 from one-way ANOVA followed by the Tukey *post hoc* test between CCO and OCO treatments.

Following the observations of GSH, GSSG, ascorbic acid, and pyroglutamate as OCO-responsive metabolites in the multivariate model of the hepatic metabolome (Figure 3 and Table 3), targeted LC-MS and biochemical analyses were conducted to evaluate the metabolites and genes related to GSH metabolism. Both GSH and GSSG in the liver were increased by feeding OCO (Figure 5A,B). However, the GSH/GSSG ratio was decreased by OCO (Figure 5C). In addition, hepatic pyroglutamate, another oxidative stress-responsive metabolite, was increased by feeding OCO (Figure 5D), while ascorbic acid tended to decrease after OCO feeding (Figure S1). Hepatic genes expression of the key enzymes in GSH synthesis, reduction, and antioxidant response including *GCLC*, *GSR*, *GPX1*, *GSTA*, and *MGST1* were elevated by dietary OCO (Figure 5E). The metabolites in the transsulfuration pathway were further quantified since it generates cysteine as a precursor for GSH synthesis. Both cystathionine and SAH in the liver were decreased by OCO treatments (Figure S2A,B), while SAM was not significantly affected (Figure S2C).

### 3.5. Effects of OCO on Threonine Catabolism

**Figure 6:**
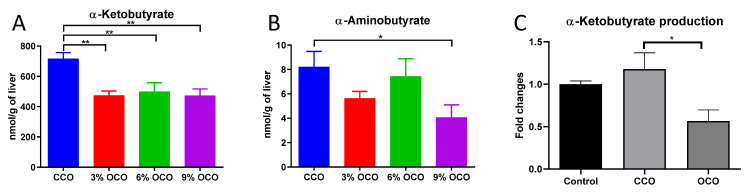
Effects of dietary concentrations of oxidized corn oil (OCO) on the hepatic metabolites of threonine catabolism. (**A**) Concentrations of α-ketobutyrate. (**B**) Concentrations of α-aminobutyrate. (**C**) Production of α-ketobutyrate after the in vitro incubation of pig liver homogenate with the polar extracts of control corn oil (CCO) and OCO. The extraction solution was used as the control. The production rate of α-ketobutyrate in the control was artificially set as 1, and the production rates in the coincubation of CCO and OCO extracts with pig liver homogenate samples were expressed as the fold of changes compared to the control. Value = mean ± SEM (n = 3 replicates per reaction). * *p* < 0.05 and ** *p* < 0.01 from one-way ANOVA followed by the Tukey *post hoc* test between the CCO and OCO dietary treatments.

The OCO-elicited increase in serum threonine concentration was explored by examining threonine catabolism, including the reactions catalyzed by TDG and TDH, by targeted metabolite analysis and in vitro reactions. Feeding OCO had little effect on the TDG pathway because OCO did not affect the enzymatic activity of TDG (Figure S3) nor the concentration of hepatic glycine, which is the downstream product of TDG (Table S5). In contrast to TDG, the TDH pathway was likely inhibited because the concentrations of hepatic α-ketobutyrate and α-aminobutyrate, two downstream metabolites in TDH pathway, were decreased by feeding OCO (Figure 6A,B). The production of α-ketobutyrate from the in vitro incubations of pig liver homogenate with the hydrophilic extracts of CCO and OCO was further examined. The results showed that the rate of α-ketobutyrate production from threonine by the liver homogenate was decreased by the co-incubation of the OCO extract in comparison with the co-incubation of the CCO extract (Figure 6C), indicating that the hydrophilic compounds in OCO are capable of inhibiting the enzyme activity of TDH directly.

## 4. Discussion

**Figure 7:**
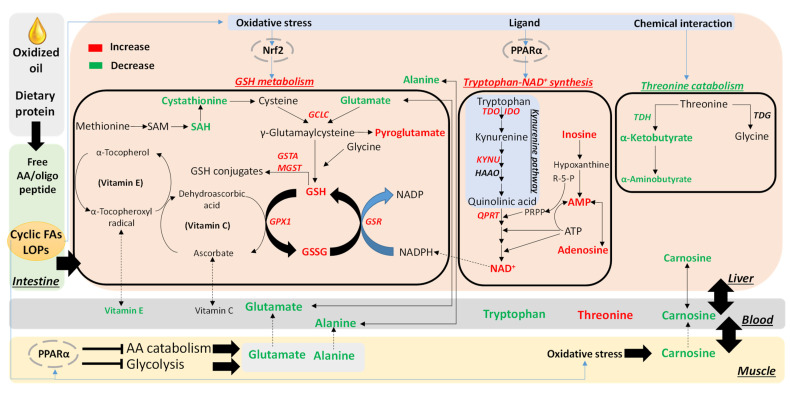
Summary of oxidized corn oil (OCO)-elicited metabolic changes in amino acid metabolism. The activation of tryptophan-NAD^+^ biosynthesis, the expansion of glutathione metabolism, and the inhibition of threonine catabolism, in the liver, as well as the decreases in glucogenic amino acids (alanine and glutamate) in serum and liver, were identified as the most prominent changes in amino acid metabolism after dietary OCO consumption. These selective changes were likely mediated by PPARα activation, oxidative stress, and Nrf2-mediated antioxidant responses in the liver and muscle. Many of these metabolic events may possess protective functions and might contribute to the tolerance of nursery pigs to the exposure of oxidized lipids.

Amino acids are the building blocks of protein and the sources of functional molecules in energy metabolism, the antioxidant system, neurotransmission, endocrine signaling, and many other biological activities [29]. Changes in amino acid homeostasis and metabolism, especially for indispensable amino acids, could have expansive impacts on biochemical and physiological events in growth and health of animals. In the current study, the effects of feeding oxidized lipids on amino acid homeostasis in young animals were evaluated by feeding OCO to nursery pigs and conducting a metabolomics-guided biochemical analysis. The most prominent changes, including the activation of tryptophan-NAD^+^ biosynthesis, the expansion of glutathione metabolism, and the inhibition of threonine catabolism, can be considered as protective and defensive events in amino acid metabolism that partially ameliorate oxidative stress and metabolic changes caused by feeding OCO in nursery pigs (Figure 7). The causes and significance of these altered metabolic activities by feeding OCO are summarized and discussed as follows.

### 4.1. Activation of Tryptophan-NAD^+^ Biosynthesis as a Protective Metabolic Event against Dietary Oxidized Lipids

In addition to being used in protein synthesis, tryptophan and its metabolites have diverse physiological and metabolic functions including the regulation of appetite, stress responses, immunity, mood, and behaviors [30]. Supplying adequate dietary tryptophan, which is an indispensable amino acid, is critical for achieving optimal growth performance and health of pigs, especially for young piglets [31]. The National Research Council (NRC) recommendation for the dietary tryptophan requirement ranges from 0.11 to 0.27% of diet for pigs in different growth stages [32]. Among its non-proteinogenic usage in the body, over 95% of dietary tryptophan is metabolized in the liver to produce NAD^+^ [30]; 1–2% is utilized to synthesize serotonin, which is a neurotransmitter with regulatory functions in appetite and gastrointestinal motility, with the remaining tryptophan being degraded into indole metabolites by the gut bacteria [33]. Therefore, the activation of tryptophan-NAD^+^ biosynthesis, which is the dominant catabolic pathway of tryptophan, can readily explain the decrease in serum tryptophan and the increase in hepatic NAD^+^ observed in this study. Moreover, it may also provide insight on the lack of changes in the hepatic concentration of tryptophan because the transport system responsible for uptake of tryptophan into hepatocytes is closely related to the activity of hepatic *TDO*, an essential enzyme in tryptophan-NAD^+^ biosynthesis [34]. The observed increase in *TDO* expression may facilitate the transport of tryptophan from the systemic circulation to the liver, which maintains hepatic concentration in the liver but decreases its serum concentration.

Nicotinamide and adenine dinucleotide, as the two structural moieties of NAD^+^, and are derived from the kynurenine pathway of tryptophan metabolism and the purine nucleotide metabolism, respectively [35]. The untargeted analysis of hepatic metabolome revealed that OCO treatments increased AMP, adenosine, and inosine, which are the intermediate metabolites in purine nucleotide metabolism and closely related to phosphoribosyl pyrophosphate (PRPP) and ATP, two cofactors that supply ribose and purine base to NAD^+^, respectively [36]. These observations indicate that dietary OCO promoted the production of adenosine and decreased purine degradation to facilitate NAD^+^ production (Figure 7). More importantly, the gene expression analysis revealed that feeding OCO induced the hepatic enzymes in kynurenine pathway. The kynurenine pathway is transcriptionally regulated by PPARα [37]. Because the peroxidation of vegetable oils is known to produce the agonists of PPARα, such as cyclic fatty acids [10,38], it is not surprising that the kynurenine pathway was activated by feeding OCO in this study (Figure 7). As a ubiquitous nuclear receptor responsive to diverse nutritional and environmental factors [39], PPARα variants across animal species differ in their sensitivity to ligand exposure, which has led to the classification of proliferating species with stronger responses to agonists, such as rodents, and non-proliferating species with less robust responses, such as pigs, monkeys, and humans [40,41]. Our previous observation of the activation of hepatic tryptophan-NAD^+^ pathway was in the mice fed 7% oxidized soybean oil, in which hepatomegaly and increases in dicarboxylic acid metabolites, which are two markers of peroxisome proliferation, were also observed [24]. However, the results from our current study of feeding 9% OCO in nursery pigs showed no hepatomegaly, as indicated by the hepatosomatic index [25], nor changes in dicarboxylic acid metabolites (data not shown), which confirms the status of pigs as a non-proliferating species. Nevertheless, our results also showed that PPARα-mediated activation of tryptophan-NAD^+^ metabolism can occur after feeding oxidized lipids to non-proliferating species, which include humans, and affect their tryptophan metabolism.

The activation of tryptophan-NAD^+^ biosynthesis has diverse consequences. In addition to being the crucial proton and electron carriers in numerous catabolic and anabolic redox reactions and energy transfer pathways, NAD^+^ and its downstream metabolites, including NADH, NADP^+^, and NADPH, have important protective functions against metabolic chemical challenges and oxidative stress [42]. A direct protective effect in nursery pigs could be increased detoxification of reactive lipidic aldehydes from OCO because NAD^+^/NADH are the cofactors of aldehyde dehydrogenases (ALDHs), which are responsible for converting aldehydes to less reactive carboxylic acids [43]. NADPH is also the terminal antioxidant in cells due to its role in recycling GSSG to GSH through GSR, as well as for converting other oxidized antioxidants back to their reduced forms [44,45]. Furthermore, LOPs, especially reactive aldehydes in oxidized oils, can cause DNA damage and mutagenic effects through their reactions with DNA [46]. When DNA damage occurs, poly (ADP-ribose) polymerase 1 (PARP1) repairs the damage using NAD^+^ as substrate for poly (ADP-ribose) formation [47]. All these NAD^+^-derived effects contribute toward the maintenance of redox balance and the protection against LOP cytotoxicity after the dietary consumption of oxidized lipids (Figure 7). At the same time, reduced supply of free tryptophan for protein and serotonin may also negatively affect the growth and feed intake. Further investigation through the augmentation of tryptophan supplementation in feeding will reveal more insights on the essentiality of tryptophan in dietary exposure of oxidized lipids.

### 4.2. Expansion of GSH Metabolism as a Protective Metabolic Event against Dietary Oxidized Lipids

The occurrence of oxidative stress is an expected consequence of the consumption of high amounts of oxidized lipids and has been widely reported in pig nutrition studies [5,25]. Decreased serum α-tocopherol was observed in our previous analyses of the pigs fed these OCO diets [25], which may be a direct consequence of scavenging LOPs from OCO. Results from our current study further showed the decrease in GSH/GSSG ratio, which is a marker of redox balance [48], a trend for decreased hepatic ascorbic acid, and decreased carnosine, which is a dipeptide antioxidant [49,50], in both serum and the liver, as the additional indicators of OCO-induced oxidative stress. In addition to these negative impacts, feeding OCO also increased both GSH and GSSG in the liver, indicating the expansion of glutathione metabolism [51]. This conclusion was supported by the increase in hepatic pyroglutamate, which is a degradation metabolite of γ-glutamylcysteine, a crucial precursor in GSH biosynthesis [52]. Moreover, the enzymes in GSH metabolism were transcriptionally upregulated by feeding OCO diets. Among these enzymes, GCLC catalyzes the initiation reaction of *de novo* GSH synthesis by converting glutamate and cysteine to γ-glutamylcysteine [53]; *GPX1* reduces lipid hydroperoxides and hydrogen peroxide to alcohols and water, respectively, and simultaneously converts GSH to GSSG; GSR is responsible for converting GSSG back to GSH; GSTs (GSTA and mGST1) conjugate GSH with electrophiles [54]. The gene expression levels of these enzymes can be induced by ROS and electrophile exposures through the regulation of transcriptional factors, including NF-E2 related factor 2 (Nrf2), activator protein-1, and nuclear factor kappa B (NF-κB) [54,55,56]. Among these transcriptional factors, Nrf2 is especially sensitive to ROS and electrophiles and highly responsive for transcriptional upregulation of GSH metabolism through Keap1, which is the anchoring and redox sensing protein partner of Nrf2 in cytosol, and the antioxidant response element (ARE), which is the binding site of Nrf2 in the 5’ promoter sequence of these GSH-related genes [57]. The observed stimulation of GSH metabolism-related genes and elevated concentration of GSH in the current study indicate that while nursery pigs underwent OCO-triggered oxidative stress, they also have a robust antioxidant system that is capable of elevating GSH metabolism to protect against ROS and partially mitigate OCO-induced toxicities (Figure 7). This conclusion is supported by the limited adverse effects of OCO consumption on the growth performance of the pigs in this study [25]. Further investigation on the GSH-related amino acid metabolism in the nursery pigs under growth-suppressing treatments will reveal more insights on the balance between oxidative stress and antioxidant response in dietary exposure of oxidized lipids.

### 4.3. Potential Protective Effects of Decreased Threonine Catabolism against Dietary Oxidized Lipids

Threonine is a limiting indispensable amino acid in swine diets, especially when feeding wheat- or corn-based diets to nursery pigs [58]. Increased availability of free threonine in the circulation may provide nursery pigs with nutritional and physiological benefits in lipid metabolism, cell proliferation, and differentiation [59]. More importantly, threonine is extensively used for mucin synthesis, which is important for nutrient digestion and absorption as well as immune function in the intestine [60,61].

TDG and TDH are two enzymes responsible for threonine catabolism in pigs and other mammals [62]. The catalytical activities of these two enzymes and their metabolites in the liver were analyzed to determine the causes that may be attributed to the observed increase of threonine concentration in the serum free amino acid pool. Feeding OCO did not affect the enzymatic activity of hepatic TDG nor the concentrations of serum and hepatic glycine, which is a product of TDG [28]. In contrast, feeding OCO negatively affected the enzymatic activity of hepatic TDH because the concentrations of α-ketobutyrate and α-aminobutyrate, two downstream products of TDH-mediated reactions, were decreased by OCO. The TDH contains pyridoxal-5’-phosphate as a cofactor, which is bound to a lysine residue in TDH through a Schiff base [63]. This bond is susceptible to the interference of aldehydes. In fact, the hydrophilic extract of OCO, which contains reactive aldehydes from lipid peroxidation, significantly reduced α-ketobutyrate production from the in vitro incubation of liver homogenate with threonine, indicating that the reactive aldehydes in OCO may directly interact with TDH and inhibit its catalytic activity (Figure 7). Furthermore, hepatic uptake of threonine and hepatic TDH activity have been shown to positively correlate with the concentrations of glucogenic amino acids, mainly alanine and glutamate, in blood [64]. It is possible that the observed decrease in alanine and glutamate by feeding OCO may also reduce hepatic threonine uptake, leading to a greater concentration of threonine in the circulation. More investigations are needed to elucidate these potential mechanisms.

### 4.4. Correlations between Serum and the Liver on Decreased Alanine and Glutamate

The serum free amino acid pool is derived from nutrient intake and endogenous amino acid metabolism. Examination of blood amino acids is an effective approach to assess nutritional status and metabolic events. Decreased serum free amino acids have been observed in malnutrition or circumstances of insufficient protein intake [65,66]. Profiling serum free amino acids have been widely used for screening metabolic diseases in humans [67]. Measuring serum free amino acids has been conducted in various animal nutrition studies. However, the correlations of serum amino acid concentrations with the status of free amino acids in organs or tissues have been rarely explored. In the current study, robust correlations between serum and liver concentrations of alanine, glutamate, and proline were observed. Moreover, both alanine and glutamate in serum and liver were decreased by feeding OCO. These correlations were not unexpected because high-flux interorgan transports occur for alanine and glutamate, which, together with glutamine, are the major carriers of nitrogen and carbon in blood for urea and glucose production in the liver, respectively [68]. Muscle is a major contributor to the presence of these amino acids in blood [69]. In muscle, alanine mainly originates from anaerobic glycolysis, which produces pyruvate, while glutamate comes from the transamination reactions between free amino acids and α-ketoglutarate in amino acid catabolism. Interestingly, PPARα activation has been shown to suppress both glucose utilization, including both glucose uptake and glycolysis [70], and amino acid catabolism in muscle and liver [71]. Therefore, PPARα activation may contribute to the observed changes in alanine and glutamate concentrations observed in the current study (Figure 7). Further studies on amino acids and associated metabolites in muscle may provide more insights into the influences of oxidized lipids on amino acid metabolism in muscle.

### 4.5. Tolerance to Oxidized Lipid Feeding and Protective Amino acid Metabolism

Our previously reported results [25] showed a lack of significant adverse effects on growth, feed intake, cardiac health, and hepatosomatic index in nursery pigs from dietary inclusion of 9% OCO from 12 h of heating at 185 °C. This tolerance from adverse effects is not an outlier among many trials where oxidized lipids are fed to swine and broiler chickens. In fact, results from a recent meta-analysis of 65 observations from feeding oxidized lipids to swine and poultry showed that while 27 of these observations showed a significant decrease in ADG, 33 observations showed no effect on growth performance measures [72]. The causes underlying tolerance and susceptibility to oxidized lipids have not been fully elucidated. Nevertheless, physiological conditions of pigs and chemical properties of oxidized lipids are expected to contribute to these different responses. The protective amino acid metabolism events that were identified in this study should be an important physiological condition contributing to the tolerance of nursery pigs to oxidized lipids. As for the chemical properties of oxidized lipids, p-anisidine value (AnV) as well as unsaturated aldehydes, which are the main contributors of AnV, stand out among multiple parameters of lipid oxidation as the potential predictors because of their robust negative correlations with the growth performance of production animals [7,73]. In the case of AnV, when feeding six thermally oxidized soybean oils (OSO) from different heating conditions, the ADG and gain: feed (G:F) of nursery pigs were compromised by feeding oils with an AnV greater than 172, but not by the oils with an AnV of less than 33 [7]. Moreover, a recent feeding trial using an OCO with an AnV of 30 also did not significantly decrease the growth performance of nursery pigs [74]. However, feeding the oxidized oils with the AnVs between 33 and 172 led to inconsistent observations. The current feeding trial using the OCO with an AnV of 138 did not significantly affect the growth of nursery pigs [25]. In a separate feeding trial, the performance of nursery pigs was compromised by the OSO with an AnV of 149 but not by another OSO with an AnV of 159 [73]. Further studies are needed to establish whether a consumption threshold based on AnV, together with other parameters of oil oxidation and animal condition, could be established for feeding oxidized lipids to pigs and also to understand the correlations and interactions among the AnV of oxidized lipids, amino acid metabolism, and growth performance.

## 5. Conclusions

Overall, the metabolomics-based biochemical analysis used in this study revealed that feeding OCO to nursery pigs induced diverse as well as selective changes in amino acid metabolism, with protective functions against LOPs and LOPs-elicited changes in redox homeostasis and nutrient metabolism. Many of these OCO-responsive metabolic events, including the activation of tryptophan-NAD^+^ biosynthesis, the expansion of glutathione metabolism, and the inhibition of threonine catabolism, are likely regulated by transcriptional factors, such as PPARα and Nrf2, and could be responsible for the tolerance of nursery pigs to OCO. These results can serve as the knowledge foundation for further exploring the usage of amino acid metabolism as a target of intervention for protecting pigs and other production animals against dietary exposure to oxidized lipids.

## Figures and Tables

**Table 1:** Effects of oxidized corn oil (OCO) on serum free amino acids and carnosine. The statistical analysis was conducted by one-way ANOVA among control corn oil (CCO) and OCO treatments followed by Tukey’s post hoc test, in which the CCO was set as the control and compared with 3%, 6%, and 9% OCO treatment groups, respectively.

	Concentration (µM)					Percentage (%) ^#^				
Metabolite	CCO	3% OCO	6% OCO	9% OCO	*p*-Value	CCO	3% OCO	6% OCO	9% OCO	*p*-Value
Ala	318.28 ^a^	286.11 ^ab^	301.56 ^ab^	254.21 ^b^	**0.04**	7.37	6.87	7.35	6.50	0.16
Arg *	116.35	124.96	117.11	108.43	0.46	2.66	3.03	2.85	2.74	0.24
Asn	83.96	68.88	71.28	72.15	0.84	1.88	1.67	1.71	1.74	0.94
Asp	25.98	22.64	23.49	20.19	0.45	0.59	0.54	0.57	0.51	0.67
Cit	39.06	41.38	42.73	42.21	0.96	0.91	0.98	1.05	1.09	0.78
Gln	453.09	381.13	411.00	390.04	0.18	10.45	9.24	10.02	10.03	0.51
Glu	474.78 ^a^	348.01 ^ab^	397.11 ^ab^	310.29 ^b^	**0.02**	10.89 ^a^	8.36 ^ab^	9.65 ^ab^	7.89 ^b^	**0.04**
Gly	1073.68	1073.73	1036.56	1018.71	0.85	24.99	25.85	25.14	26.28	0.82
His *	51.63	55.21	49.85	48.08	0.47	1.18	1.34	1.22	1.20	0.67
Iso/Leu *	204.66	197.58	209.91	180.88	0.40	4.68	4.77	5.10	4.58	0.38
Lys *	149.08	132.75	124.84	154.35	0.62	3.40	3.23	2.97	3.85	0.33
Met *	22.84	19.34	22.80	23.94	0.66	0.52	0.47	0.55	0.59	0.58
Orn	60.10	63.74	61.31	67.11	0.80	1.36	1.53	1.48	1.71	0.15
Phe *	124.66	131.40	124.30	114.91	0.36	2.87	3.21	3.04	2.92	0.36
Pro	278.39	269.86	275.09	253.78	0.50	6.43	6.50	6.69	6.46	0.72
Ser	215.06	233.19	205.54	219.91	0.71	4.94	5.67	4.95	5.57	0.25
Tau	228.05	227.05	181.05	204.54	0.85	5.18	5.47	4.28	5.23	0.80
Thr *	118.64	150.78	147.48	146.60	0.44	2.69	3.63	3.55	3.67	**0.06**
Trp *	43.06 ^a^	34.34 ^ab^	35.80 ^ab^	30.98 ^b^	**0.04**	0.99	0.84	0.86	0.78	**0.10**
Tyr	46.75	47.14	56.25	60.60	0.33	1.05	1.13	1.38	1.46	0.11
Val	215.99	232.79	230.90	203.55	0.60	4.95	5.63	5.59	5.15	0.38
Total	4344.06	4141.98	4125.95	3925.44	0.40					
Carnosine	14.56	11.53	9.01	9.3	**0.01**					

*: Indispensable amino acids. ^#^: Relative abundances of individual amino acids are calculated as the percentages of total amino acids. ^a,b^: Different superscripts in a row indicate significant differences between treatments (*p* < 0.05).

**Table 2:** Confirmed oxidized corn oil (OCO)-responsive amino-containing metabolites in serum. The metabolites were detected in positive mode after the DC derivatization ([M+DC]^+^) and identified as the markers from multivariate modeling.

Ions	Modes of Ion Detection	*m*/*z* of Detection	Identity	Formula	∆ppm	Database ID	Effects of OCO
I_s_	[M+DC]^+^	323.1053	Alanine *	C_3_H_7_NO_2_	4.0	HMDB0000161	↓
II_s_	[M+DC]^+^	381.1110	Glutamate *	C_5_H_9_NO_4_	2.6	HMDB0060475	↓
III_s_	[M+DC]^+^	438.1478	Tryptophan *	C_11_H_12_N_2_O_2_	2.3	HMDB0030396	↓
IV_s_	[M+DC]^+^	460.1648	Carnosine *	C_9_H_14_N_4_O_3_	0.4	HMDB0000033	↓
V_s_	[M+DC]^+^	353.1160	Threonine *	C_4_H_9_NO_3_	3.1	HMDB0000167	↑

*: confirmed by authentic standards.

**Table 3:** Identification of hepatic metabolites changed by oxidized corn oil (OCO) feeding. The metabolites were detected in negative mode ([M−H]^−^), positive mode ([M+H]^+^), or positive mode after the DC derivatization ([M+DC]^+^), and identified as the markers from multivariate modeling.

Ions	Modes of Ion Detection	*m*/*z* of Detection	Identity	Formula	∆ppm	Database ID	Effects of OCO
I_L_	[M-H]^−^	175.0236	Ascorbic acid *	C_6_H_8_O_6_	−4.5	HMDB0000044	↓
II_L_	[M+DC]^+^	323.1055	Alanine *	C_3_H_7_NO_2_	−3.4	HMDB0000161	↓
III_L_	[M−H]^−^ [M+H]^+^	346.0551 348.0691	AMP *	C_10_H_14_N_5_O_7_P	−0.6 −5.2	HMDB0000045	↑
IV_L_	[M−H]^−^ [M+H]^+^	611.1444 613.1590	GSSG *	C_20_H_32_N_6_O_12_S_2_	0.7 −1.3	HMDB0003337	↑
V_L_	[M−H]^−^	306.0758	GSH *	C_10_H_17_N_3_O_6_S	−4.8	HMDB0000125	↑
VI_L_	[M+H]^+^	664.1165	NAD *	C_21_H_27_N_7_O_14_P_2_	−0.1	HMDB0000902	↑
VII_L_	[M+H]^+^	130.0499	Pyroglutamate *	C_5_H_7_NO_3_	−2.5	HMDB0000267	↑

*: confirmed by authentic standards.

## Data Availability

Data are contained within the article and the supplementary material.

## Supplementary Materials

The following supporting information can be downloaded at: https://www.mdpi.com/article/10.3390/metabo13010103/s1, Figure S1: Concentration of hepatic ascorbic acid in control corn oil (CCO)- and oxidized corn oil (OCO)-treated pigs. Figure S2: Effects of oxidized corn oil (OCO) on the concentrations of hepatic metabolites in the transsulfuration pathway. Figure S3: Hepatic TDG enzyme activity. Table S1: Source of biochemicals and reagents used in chemical analysis, LC-MS analysis, structural confirmation, and quantification. Table S2: LC-MS data acquisition condition in a 10 min run. Table S3: The sequence of primers used in the real-time PCR analysis. Table S4: Additional oxidized corn oil (OCO)-responsive amino-containing serum metabolites with tentative identities. Table S5: Effects of oxidized corn oil (OCO) on hepatic free amino acids. Table S6: Additional oxidized corn oil (OCO)-responsive hepatic metabolites with tentative identities.
