# Supplementary material for: Identification of Protective Amino Acid Metabolism Events in Nursery Pigs Fed Thermally Oxidized Corn Oil
Source: Metabolites. 2023 Jan 8;13(1):103. doi: 10.3390/metabo13010103 (PMC9866068; doi:10.3390/metabo13010103)

Article

Identification of Protective Amino Acid Metabolism Events in Nursery Pigs Fed Thermally Oxidized Corn Oil

Yue Guo [1], Lei Wang [1], Andrea Hanson [2,†], Pedro E. Urriola [2], Gerald C. Shurson [2] and Chi Chen [1,2,*]

1 Department of Food Science and Nutrition, University of Minnesota, 1334 Eckles Ave., St. Paul, MN 55108, USA
2 Department of Animal Science, University of Minnesota, 1364 Eckles Ave., St. Paul, MN 55108, USA
* Correspondence: chichen@umn.edu; Tel.: +1-612-624-7704; Fax: +1-612-625-5272
† Current address: Professional Swine Management, LLC, 303 North 2nd St., Carthage, IL 62321, USA.

SUPPLEMENTARY DATA

Table S1. Source of chemicals and reagents used in biochemical analysis, LC-MS analysis, structural confirmation, and quantification.

**Chemicals and reagents**	**Vendor**
Acetone (LC-MS grade), Acetonitrile (LC-MS grade), Ammonium formate, Chloroform, Formic acid (LC-MS grade), Isopropanol (LC-MS grade), Water (LC-MS grade)	Fisher Scientific (Houston, TX)
Adenosine, Adenosine-5-monophosphoric acid (AMP), 2-Hydrazinoquinoline (HQ), Inosine, Triphenylphosphine (TPP)	Alfa Aesar (Ward Hill, MA)
2-2′-Dipyridyl disulfide (DPDS)	MP Biomedicals, LLC (Irvine, CA)
d_4-Acetic acid, Amino acid standards, α-Amino-n-butyric acid, n-Butanol, Dithiothreitol, Dansyl chloride (DC), Glutathione oxidized (GSSG), Glutathione reduced (GSH), α-Ketobutyric acid, β-Nicotinamide adenine dinucleotide hydrate (NAD), Potassium phosphate monobasic (KH_2PO_4), Sodium carbonate (Na_2CO_3)	Sigma-Aldrich (St. Louis, MO)
Pyridoxal 5-phosphate	TCI America (New Brunswick, NJ)
L-Threonine	Acros Organics (Morris Plains, NJ)
d_5-Tryptophan	Cambridge Isotope Laboratories (Tewksbury, MA)

Table S2: LC-MS data acquisition condition in a 10-minute run.

Target compounds	Column type	Mobile phase	MS detection mode
Hydrophobic metabolites & Amino acids (dansylated)	BEH C18	A: 0.1% formic acid in H_2O<br>B: 0.1% formic acid in ACN	Positive and negative
Fatty acids (HQ derivatization)	BEH C18	A: 2 mM NH_4OAc in water with 0.05% CH_3COOH<br>B: 2 mM NH_4OAc in 95% ACN and 5% H_2O with 0.05% CH_3COOH	Positive
Hydrophilic metabolites	BEH Amide	A: 0.1% formic acid in H_2O<br>B: 0.1% formic acid in ACN	Positive and negative

Table S3: The sequence of primers used in the real-time PCR analysis.

Gene	Forward primer (from 5′ to 3′)	Reverse primer (from 5′ to 3′)
TDO2	GAGAGTCCCTTACAACAGGAGAC	CATGTGGCTCTAAACCTGGAGT
IDO2	AGACGCAGCCCAAAGAGGTT	ACGGATGCTTTCTCCCCCAG
KYNU	ACCTGCCATCACAAAAGCTGG	TAGGAGCACCAGCAGGCAAA
HAAO	AGCAGCCACAGGGTATGTCC	CTTGGCAGCAGCACACGTAG
QPRT	GGAGCGGGTGGCCCTTAATA	GGTCGTACCTGTGGGAGGTG
TDG	TGGTGCCCATAGCAGAGTCG	TTGGGCTCTGGAACCTTGGG
GPX1	GGGGAGATCCTGAATTGCCT	GAAGAGCGGGTGAGCATTTG
GSR	TGCGTGAATGTCGGATGTGT	GTGTTCAGTCGGCTCACGTA
GSTA1	AGACTCAAGACCTGGATAAGT	AGGCCACCTTGGCATCTTTT
MGST1	AGCCCAGAATGACCTTG	ACGATTTGGCTGGGGAAGG
GCLC	CTGCCTGAGTACAAGCCCAA	CTCCTGTGCCGGATGTTTCT
ACTB	ATCGCCGACAGGATGCAGAA	ATCGCCGACAGGATGCAGAA

Table S4. Additional oxidized corn oil (OCO)-responsive amino-containing serum metabolites with tentative identities.

Ions	Modes of Ion detection	*m/z* of detection	Identity	Formula	Δppm	Database ID	Effects of OCO
VI_s	$[M+DC]^+$	363.1003	Pyroglutamate*	$C_5H_7NO_3$	-3.3	HMDB0000267	↓
VII_s	$[M+DC]^+$	362.1181	Hydrouracil*	$C_5H_8N_2O_2$	1.7	HMDB0000076	↓
VIII_s	$[M+H]^+$	520.3399	LysoPC(18:2)*	$C_{26}H_{50}NO_7P$	-0.8	HMDB0061700	↓
IX_s	$[M+DC]^+$	379.1682	Acetylcholine*	$C_7H_{15}NO_2$	-2.6	HMDB0000895	↑
X_s	$[M+DC]^+$	321.1262	4-Aminobutyraldehyde*	C_4H_9NO	-3.4	HMDB0001080	↑

*: *The identities were defined by the accurate mass-based database search.*

Table S5. Effects of oxidized corn oil (OCO) on hepatic free amino acids. The statistical analysis was conducted by one-way ANOVA among control corn oil (CCO) and OCO treatments followed by Tukey's *post hoc* test, in which the CCO was set as the control and compared with 3%, 6%, and 9% OCO treatment groups, respectively. The correlations between serum and hepatic free amino acids and their significance were examined by the Pearson correlation analysis.

	Concentration (μg/g)					Pearson correlation	
Metabolite	CCO	3% OCO	6% OCO	9% OCO	*p*-value	r value	*p*-value
Ala	365.06	282.77	303.11	244.25	**0.08**	0.85	**<0.01**
Arg*	13.15	12.17	11.62	11.17	0.42	0.03	0.86
Asn	48.51	43.31	29.30	32.94	0.43	0.24	0.20
Asp	142.97	139.10	181.06	136.20	0.66	0.23	0.20
Cit	16.40	16.36	16.40	16.34	0.78	-0.13	0.47
Gln	511.72	492.27	460.03	498.40	0.84	-0.07	0.73
Glu	857.75[a]	679.85[ab]	678.47[ab]	656.06[b]	**0.04**	0.60	**<0.01**
Gly	381.55	330.16	277.69	314.18	0.15	0.13	0.47
His*	33.44	30.95	26.95	26.94	0.28	0.24	0.20
Iso/Leu*	46.28	39.06	27.22	34.37	0.47	0.28	0.13
Lys*	46.49	33.36	27.22	34.37	0.35	0.12	0.52
Met*	12.94	11.83	11.35	11.36	0.23	-0.07	0.70
Orn	18.91	17.26	14.41	15.97	0.66	0.18	0.34
Phe*	21.32	19.27	15.91	17.84	0.52	0.08	0.66
Pro	77.74	62.21	59.64	53.64	0.11	0.51	**<0.01**
Ser	183.84	154.37	126.14	145.87	0.53	0.23	0.21
Tau	375.98	317.94	225.26	575.97	0.26	0.04	0.79
Thr*	31.25	30.27	27.42	26.56	0.78	0.14	0.46
Trp*	12.94	12.71	12.66	12.71	0.97	0.11	0.53
Tyr	23.54	19.94	17.02	18.69	0.34	0.05	0.80
Val	40.26	33.67	30.27	30.55	0.38	0.01	0.97
Carnosine	25.64	22.56	21.97	22.17	**0.01**	0.30	**0.09**

*: *Indispensable amino acids.*

[a,b]: *Different superscripts in a row indicate significant difference between treatments (P < 0.05).*

Table S6. Additional oxidized corn oil (OCO)-responsive hepatic metabolites with tentative identities.

Ions	Modes of Ion detection	*m*/*z* of detection	Identity	Formula	Δppm	Database ID	Effects of OCO
VIII_L	$[M-H]^-$	133.0133	Malic acid*	$C_4H_6O_5$	-3.0	HMDB0000156	↓
IX_L	$[M+H]^+$	123.0552	Nicotinamide*	$C_6H_6N_2O$	-4.9	HMDB0001406	↓
X_L	$[M+H]^+$	137.0458	Hypoxanthine*	$C_5H_4N_4O$	-3.7	HMDB0000157	↓
XI_L	$[M-H]^+$	540.0534	Cyclic adenosine diphosphate ribose*	$C_{15}H_{21}N_5O_{13}P_2$	0.2	HMDB0249529	↑
XII_L	$[M-H]^+$	565.0474	UDP-glucose*	$C_{15}H_{24}N_2O_{17}P_2$	0.4	HMDB0000286	↑

*: *The identities were defined by the accurate mass-based database search.*

Figure S1: Concentration of hepatic ascorbic acid in control corn oil (CCO)- and oxidized corn oil (OCO)-treated pigs. Value = mean ± SEM (n = 8 pigs/group).

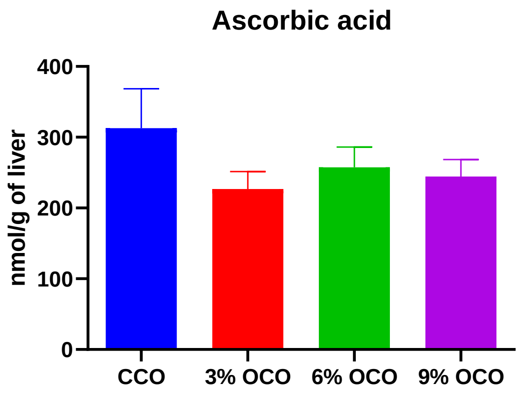

Figure S2: Effects of oxidized corn oil (OCO) on the concentrations of hepatic metabolites in the transsulfuration pathway. (**A**) Cystathionine. (**B**) SAH. (**C**) SAM. Value = mean ± SEM (n = 8 pigs/group). The statistical difference between control corn oil (CCO) and OCO treatments is labeled as * to indicate $P < 0.05$ from one-way ANOVA followed by the Tukey *post hoc* test.

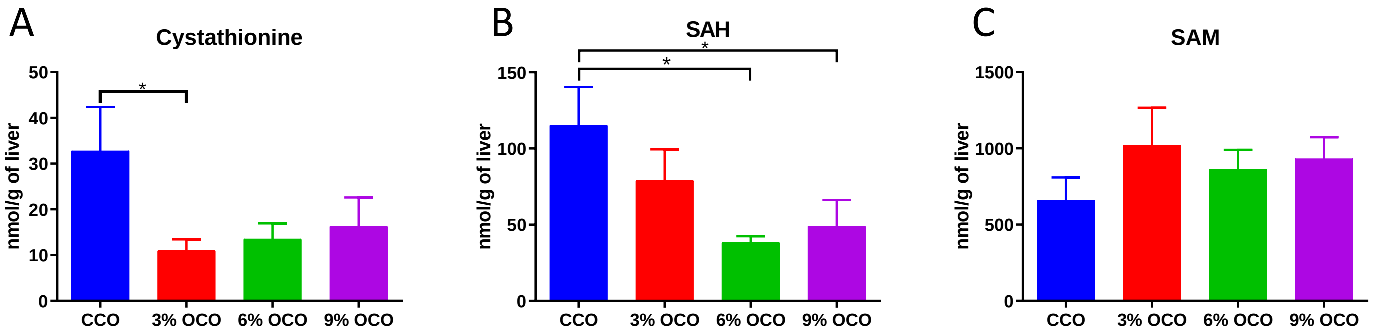

Figure S3: Hepatic TDG enzyme activity. The enzymatic activity of TDG was measured in the liver of control corn oil (CCO)- and oxidized corn oil (OCO)-treated pigs. The unit is presented as IU/g of liver tissue. Value = mean ± SEM (n = 8 pigs/group).

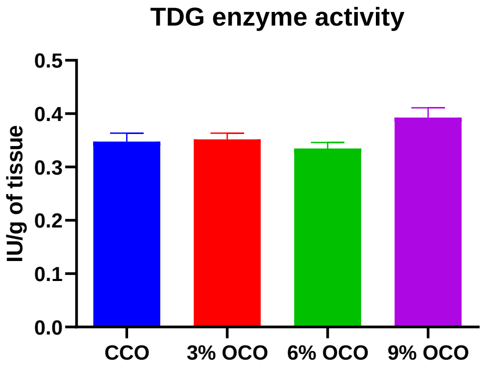

Supplement: Supplementary file 1 [file metabolites-13-00103-s001.zip › metabolites-2114090-supplementary.pdf]
